# Predicting Virtual World User Population Fluctuations with Deep Learning

**DOI:** 10.1371/journal.pone.0167153

**Published:** 2016-12-09

**Authors:** Young Bin Kim, Nuri Park, Qimeng Zhang, Jun Gi Kim, Shin Jin Kang, Chang Hun Kim

**Affiliations:** 1 Interdisciplinary Program in Visual Information Processing, Korea University, Seoul, Korea; 2 Department of Computer and Radio Communications Engineering, Korea University, Seoul, Korea; 3 School of Games, Hongik University, Seoul, Korea; East China University of Science and Technology, CHINA

## Abstract

This paper proposes a system for predicting increases in virtual world user actions. The virtual world user population is a very important aspect of these worlds; however, methods for predicting fluctuations in these populations have not been well documented. Therefore, we attempt to predict changes in virtual world user populations with deep learning, using easily accessible online data, including formal datasets from Google Trends, Wikipedia, and online communities, as well as informal datasets collected from online forums. We use the proposed system to analyze the user population of EVE Online, one of the largest virtual worlds.

## Introduction

Evolution in network technology and computing power has enabled people to interact with one another over the Internet. In the same vein, a growing number of users interact with one another and engage in economic, educational, and artistic activities in large virtual worlds (e.g., Second Life). Additionally, massively multiplayer online games (MMOGs)—e.g., World of Warcraft and EVE Online—have attracted an increasing number of active users who build communities and participate in a range of interactions [1–6].

Research on virtual worlds or online societies has been extensive. Easily accessible data from Pardus, an MMOG, enables researchers to investigate social theories in large virtual populations [7–12]. In particular, research on the structure and dynamic evolution of socioeconomic networks in virtual worlds has demonstrated significant results using diverse approaches [13–18]. Moreover, data from prior research has been analyzed from different perspectives [19–24]. In addition, research on forecasting the value of virtual currencies used in economic activity among virtual world users is currently underway [25, 26].

Previous studies focused on analyzing virtual world users based on social theories; research into user dynamics in virtual worlds is difficult to find. By contrast, extensive research on predicting real-world population fluctuations has been conducted [27–33]. Still, most researchers have adopted long-term perspectives regarding predictions of real-world population dynamics. These methods could be applied to virtual environments and combined with a range of internal components to find meaning in long-term population trends. In this study, we focus on the characteristics of virtual worlds such as MMOGs, whose rapid changes correlate with increasing or decreasing user populations [34–36].

The present paper focuses on EVE Online, one of the largest MMOG virtual worlds, which has attracted approximately 0.5 million subscribers since it was released in May 2003 [37]. We predict the daily fluctuations in the number of users of EVE Online through deep learning [38, 39], based on Google Trends [40], Wikipedia usage [41, 42], online forum usage [43] and sentiment data from online forum postings. The proposed method achieved a certain level of success in predicting daily user population fluctuations by drawing on easily available data closely associated with virtual worlds.

## Methods

### System overview

**Fig 1:**
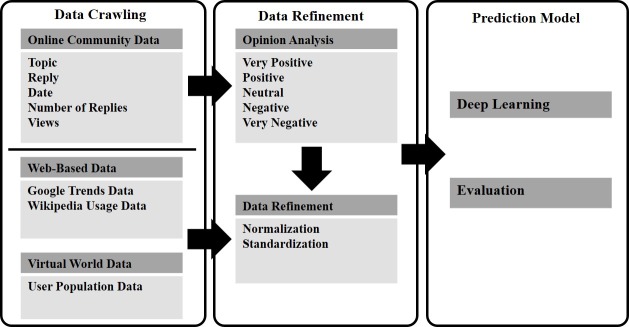
System overview.

For the proposed system, we collected data associated with MMOG EVE Online and tagged each posting and reply in its online forum with a positive or negative sentiment value. Based on this data and deep learning [38], we created a model for predicting fluctuations in the number of users (Fig 1).

### Data collection

To generate prediction models, we selected data considered to be associated with an increase or decrease in the number of EVE Online users. The data selected was easily accessible online and was gathered from three sources: Google Trends, Wikipedia, and EVE Online forums [40–43].

Google Trends measures search interest in keywords searched on Google over a given period on a scale from 1 to 100. Google Trends data are widely used to analyze relevant phenomena in a variety of fields [44–49]. We gathered Google Trends data about the keyword “EVE Online” for this paper.

Second, Wikipedia usage data [41, 42] was gathered, showing the number of page views for certain keywords on a given day. These data are also widely used to analyze phenomena on the Internet [46, 50, 51]. Again, we gathered the Wikipedia page data relevant to “EVE Online”.

We also gathered data from EVE Online forums [43], which are used by community members to upload postings and exchange opinions regarding topics of common interest [25, 52–54]. Therefore, online forums provide good sources for monitoring the daily responses of many users to certain MMOGs. Communities or forums are widely used in MMOGs for information exchange [52]. According to one study, EVE Online linked its forums with economic activity among users [25], which we found to be relevant when predicting fluctuations in the current number of virtual world users. Comments and relevant replies posted by users on general discussion boards in EVE Online forums were crawled, along with post time, comment and reply post times, the number of replies to each comment, and the number of views. Replies quoting previous comments and replies were crawled to exclude overlapping sentences.

Each HTML page was crawled using Python regex to parse HTML tags and extract the number of topics, the number of replies, the dates on which the topics and replies were posted, and the URL of each topic from the general discussion boards. Based on the URLs of extracted topics, content and replies to them were also extracted. These data were saved in.json format, which was in turn converted to other formats (e.g. csv and xlsx) for different purposes. The.json files from the EVE Online forums crawled can be viewed in the supporting information. One researcher carried out this data collection on a single PC for approximately 72 hours.

We collected data over a period extending from September 12, 2011 to April 29, 2016 (see Table 1) in a manner that complies with the terms and conditions stipulated by each service. Moreover, the collected data do not include any personal information.

**Table 1:** Summary of crawled data

Source	Boundary	Data Volume
EVE Online Community	Sep. 12, 2011–Apr. 29, 2016	1,156,608 User Replies
		37,418 User Threads
Google Trends (EVE Online)	Sep. 12, 2011–Apr. 29, 2016	1,692 Google Trends Values (1 value per day)
Wikipedia Usage (EVE Online)	Sep. 12, 2011–Apr. 29, 2016	1,692 Wikipedia Usage Values (1 value per day)

### Tagging user comment data and correlation analysis

We tagged the collected user comment data with positive or negative sentiment values. Previous studies have mostly focused on classifying user comments in certain fields. Emoticons, neologisms, and ungrammatical expressions are frequently seen on Internet forums. C.J. Hutto and Eric Gilbert [55] proposed an algorithm (VADER) to complement such informal expressions and suggested a method of analyzing social media text using a rule-based model. The online forums of interest here are comparable to social media text. Thus, we utilized the VADER algorithm to tag user comment data from the forums.

VADER normalizes negative and positive sentiment on a scale of -1 to 1. A comment was tagged as very negative, negative, positive or very positive, for -1 ≤ *x* < -0.6, -0.6 ≤ *x* < -0.2, 0.2 ≤ *x* < 0.6, and 0.6 ≤ *x* ≤ 1.0, respectively (where *x* is a number). Each posting and reply was tagged (see the opinion analysis example in Table 2).

**Table 2:** EVE Online Forum Opinion Analysis Example

Opinion Criteria	Example topic sentences
Very Positive	“Pretty happy with Crucible” / “Happy New Year from CCP Games!” / “The days of "Erotica 1" are gone and I AM GLAD” / “Great job CCP!”
Positive	“Nice launcher” / “Better Missile graphics are good” / “CCP Thank you for new Capital Rats”
Neutral	“Guide to Fleet Commanding” / “Drone ships completely useless especially for new players.” / “A New Player Guide” / “Worth coming back to eve?”
Negative	“Why does Something Awful forums looks so old and terrible?” / “Back to play this terrible game terribly” / “Server crashed?” / “Awful download speed since launcher is released.”
Very Negative	“Those WTF moments” / “AFK Cloaking in System is a Terrible Mechanic” / “Verification Failure—Still Happening” / “Eve Installer not working”

We verified the correlation between tagged sentiment values and the increase or decrease in user population. Here, the Pearson correlation coefficient [56] was used to determine the correlation between data sources and the increase or decrease in user population.

**Fig 2:**
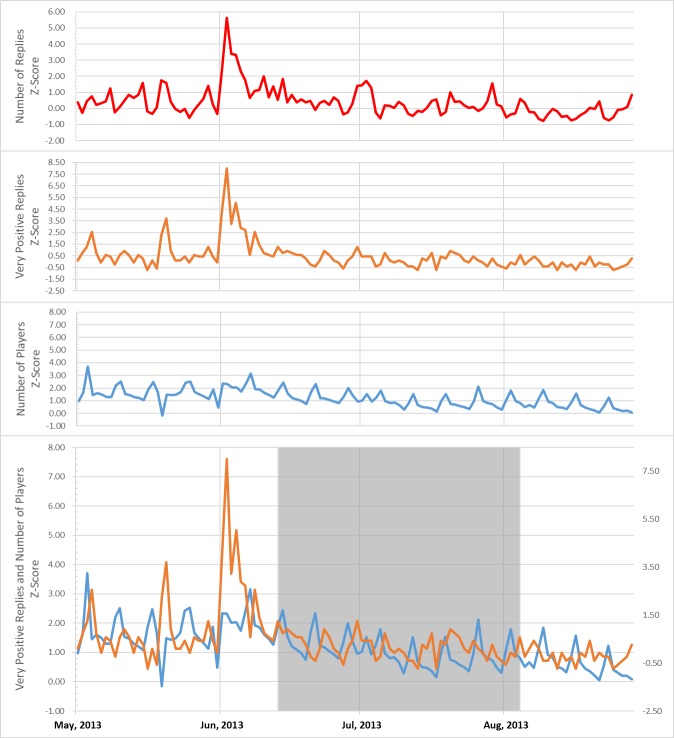
Z-scores of fluctuations in population and results of opinion analysis. Some opinions show a trend similar to that of fluctuations in the population.

As shown in Eq (1), the results of opinion analysis based on the topics and replies (VADER-based tagged values) and population fluctuations were transformed each into z-scores for standardization against those of the previous 20 days. On a given date *t* (*t* = 20 in the paper), the z-score of expected value E, denoted by $Z_{E}$, was defined as:

$$Z_{E_{t}}=\frac{E-\bar{x}(E)}{\sigma (E)}$$ (1)

where $\bar{x}(E)$ and σ(E) represent the mean and standard deviation of each item for every date with a time granularity of 1 day. Fig 2 shows an example of test results comparing the fluctuations in population and opinion analysis z-scores.

Table 3 shows the Pearson correlation coefficients between the z-scores for the opinion analysis results and the z-scores for population fluctuation.

**Table 3:** Pearson Correlation Coefficient Result

Opinion	Pearson Correlation Coefficient between results of opinion analysis and the population
Very Negative Topic	0.2547
Negative Topic	0.2422
Neutral Topic	0.2136
Positive Topic	0.2784
Very Positive Topic	**0.3037**
Very Negative Reply	**0.3178**
Negative Reply	**0.3667**
Neutral Reply	**0.3047**
Positive Reply	**0.3380**
Very Positive Reply	**0.3298**

Overall, a positive linear relationship was found. Notably, there was a clear correlation between user replies and very positive topics.

### Prediction Modeling

We used the data collected and the tagged opinion data for the model with the intent to predict fluctuations in user population based on deep learning. Deep learning is widely used for solving a range of problems [38, 39, 57–62]. Data sources have increased quantitatively and qualitatively in proportion to the history of virtual worlds. Still, little research on applying deep learning to virtual worlds for problem solving has been conducted. We developed a setting for applying deep learning based on collected data from a 4.5-year period.

First, we refined the data for the learning models. Specifically, the data gathered (as explained above) was standardized against its applicability to learning. We used long-term data and feature vectors by standardizing the data against the previous 20 days to lessen the impact of the significant changes in the range of data values over longer time periods. An example of applicable input data is shown in Table 4.

**Table 4:** Example of a deep learning dataset. The z-score $Z_{E_{t}}$ for data from the previous 20 days was used as the values A–J, which indicate the value of the sum of forum opinion on a given date. V–Z denote formal data values (number of topics, sum of replies, sum of views, Google Trends value, and Wikipedia page views) on a given date.

Data Class	Date	Opinion Data										Formal Data				
		Very positiveTopic	PositiveTopic	NeutralTopic	NegativeTopic	Very negativeTopic	Very positiveReply	PositiveReply	NeutralReply	NegativeReply	Very negativeReply	Number of Topics	Sum of Replies	Sum of Views	Google Trends Value	Wikipedia Page Views
Crawled Raw Data	Apr 02, 2016	*A*	*B*	*C*	*D*	*E*	*F*	*G*	*H*	*I*	*J*	*V*	*W*	*X*	*Y*	*Z*
Input Learning Data	Apr 02, 2016	$Z_{A_{t}}$	$Z_{B_{t}}$	$Z_{C_{t}}$	$Z_{D_{t}}$	$Z_{E_{t}}$	$Z_{F_{t}}$	$Z_{G_{t}}$	$Z_{H_{t}}$	$Z_{I_{t}}$	$Z_{J_{t}}$	$Z_{V_{t}}$	$Z_{W_{t}}$	$Z_{X_{t}}$	$Z_{Y_{t}}$	$Z_{Z_{t}}$

We built deep learning models to perform predictions based on the input data. We accumulated multiple hidden layers to learn the deep structure of the data. Here, we configured 1, 2, 3, 5, and 7 hidden layers; of these, we selected the one that returned the best prediction results. We allocated 1,500 neurons to the single hidden layer; when using 2 hidden layers, we allocated 1,024 neurons to each; when using 3 hidden layers, we allocated 1,024, 1,024, and 512 neurons; when using the 5 hidden layers, we allocated 2,048, 1,024, 1,024, 512 and 512 neurons to them; and finally, when using 7 hidden layers, we allocated 2,048, 1,024, 1,024, 1,024, 512, 512 and 512 neurons to the layers.

For the input layer, based on the input data in Table 4, we represented 15 input data as continuing vectors and allocated a different number of neurons to a different number of cumulative days used for learning (i.e., 45, 75, 105, 135, and 180 neurons were allocated to 3, 5, 7, 9, and 12 cumulative days, respectively). For the output layer, 2 neurons were allocated to represent the probabilities of population increase or decrease with the softmax function.

We implemented the model using the Google Tensorflow library [38] and accelerated the deep learning model with GPU operation (nVIDIA CUDA). The gap in the prediction values between the optimal model and other model configurations is discussed in the following section.

## Experimental Results

We performed prediction modeling by means of deep learning based on the collected and refined data, and predicted fluctuations in the EVE Online user population. We used 90% of the data from the period between September 12, 2011 and April 29, 2016 for learning and 10% for validation. The accuracy rate, F-measure, and Matthews correlation coefficient (MCC) were used to evaluate the performance of the proposed models.

**Fig 3:**
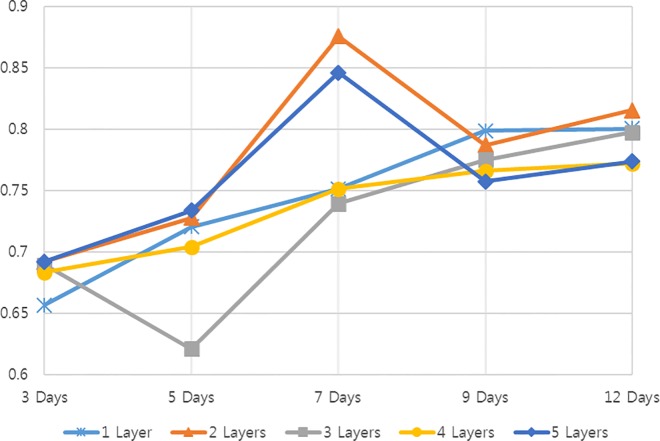
Experimental results. Values by data and layers used.

Table 5 and Fig 3 show the prediction results. The highest accuracy (87.57%) resulted from the 2-layer neural network model that used data from the previous 7 days for learning. Table 5 outlines the prediction results by layer configuration and learning data. If the number of hidden layers and days used were less than 2 and 7, respectively, learning was insufficient and prediction accuracy decreased slightly. Conversely, overfitting could occur (with the prediction accuracy failing to significantly improve) if these numbers exceeded 2 and 7, respectively.

**Table 5:** Experimental results of predicted fluctuations in the EVE Online user population

Data Set		Accuracy (%)	F1-Score	MCC
Hidden Layers	Learning Days			
1 Hidden Layer	3 Days	65.68%	0.6577	0.3098
	5 Days	72.04%	0.7199	0.4342
	7 Days	75.15%	0.753	0.5145
	9 Days	79.88%	0.8006	0.6042
	12 Days	80.06%	0.8027	0.6027
2 Hidden Layers	3 Days	69.23%	0.6933	0.3815
	5 Days	72.78%	0.7271	0.4495
	7 Days	**87.57%**	**0.8763**	**0.7517**
	9 Days	78.70%	0.788	0.5802
	12 Days	81.55%	0.8147	0.6127
3 Hidden Layers	3 Days	68.94%	0.6895	0.3756
	5 Days	62.13%	0.6213	0.2283
	7 Days	73.96%	0.74	0.4822
	9 Days	77.52%	0.7763	0.5438
	12 Days	79.76%	0.7988	0.5981
5 Hidden Layers	3 Days	68.34%	0.6844	0.3572
	5 Days	70.41%	0.7040	0.4078
	7 Days	75.15%	0.7521	0.4987
	9 Days	76.63%	0.768	0.5344
	12 Days	77.23%	0.7732	0.536
7 Hidden Layers	3 Days	69.23%	0.6935	0.408
	5 Days	73.37%	0.7337	0.4675
	7 Days	84.62%	0.8468	0.6897
	9 Days	75.74%	0.7578	0.5079
	12 Days	77.38%	0.7751	0.5386

## Discussion and Conclusion

This paper proposed a new method for predicting fluctuations in the number of users in a massive virtual world, EVE Online, with a deep-learning prediction model based on data from a variety of sources. The proposed method successfully predicted fluctuations in the number of EVE Online users based on the easily accessible data relevant to the virtual world. User comments in online forums were found to affect user actions in the virtual world.

The proposed method could be applicable to diverse fields, e.g., verifying newly added content in the creation and management of virtual worlds and solving network problems by forecasting the number of users. The proposed method could also be applied to previous findings, e.g., space and NPC management in virtual worlds [63, 64] and to the optimization of virtual currency systems [25]. In addition, it could be used to apply social science theories to virtual worlds so as to understand the large, diverse user base.

Due to the paucity of previous findings in this field, our proposal has some limitations that need to be rectified in future work. First, the data need to be enriched further for better results. Diverse types of data would increase prediction accuracy. This research was limited to using data gathered for the prediction model only by means of deep learning; more diversified data would be applicable to feature selection as well. The VADER algorithm, optimized for social media analysis, performed well in this study. However, additional analysis of sarcastic or ironic language would improve our results. Moreover, Word2Vec [65] could also potentially improve these results. Sentiment analysis using the Word2Vec-based Doc2Vec [66] focuses on user reviews, making it difficult to apply directly to the analysis of online community users. Still, it might be applicable to and improve the analysis of user comments in online communities. As all data used for learning were formal data, data refinement methods should also be improved. Furthermore, other sizable virtual worlds, e.g., World of Warcraft and Second Life, could be used for prediction.

Virtual worlds have been growing in size and diversity. Considerable research on them has already been conducted. Still, the size of the virtual world user population has not been elucidated. With ongoing improvement for wider application, the proposed method of applying data from different sources to virtual world user population dynamics will contribute to enhancing the understanding of virtual worlds and their user bases.

## Supporting Information

S1 File Results of crawling EVE Online forum (in.json format) (7Z)

S2 File Python-based crawler source code for forum data collection (ZIP)

S3 File Community usage, Google Trends values, activity on Wikipedia, sentiment analysis results, and EVE Online population data used in the experiment (in.csv format) (CSV)
